# Correspondence

**Published:** 1896-04

**Authors:** 


### CORRESPONDENCE.

EDITOR JOURNAL OF COMPARATIVE MEDICINE, ETC.

I SEE by the columns of the March number of the JOURNAL that many States, including Illinois, have a bill in preparation to present to the members at the next term of the Legislature for veterinary legislation.

The JOURNAL at all times is deeply interested in the advancement of veterinary science, and through the columns of your able JOURNAL no doubt the veterinarians could all be stirred up to work for legislation, as in unity there is strength. The Illinois State Veterinary Medical Association has now been in existence since 1885; during that time three bills have been presented to the Legislature at Springfield. The several bills referred to have all been introduced at the request of the members of the Association. Drs. Williams, Shephard, Casewell, Withers, Hughes, Baker, Trumbower, Wilson, and others of the members have done noble and arduous work to procure veterinary legislation for this State.

Although many of the members of our Association who in the past worked so earnestly and spent their time and money for legislation have left the State, the present members of the Association will again present a bill to the Legislature at the next term in Springfield.

The membership of our Association numbers less than 100; the number of members located in Chicago, 17. That is a small percentage of veterinary surgeons, graduates of recognized veterinary colleges, practising in Chicago.

In looking over the *City Directory* I find 129 names recorded as veterinary surgeons, 50 of whom I am acquainted with and know to be graduates of recognized veterinary colleges. The McKillip Veterinary College, which opened its doors in 1894 with a three-year course of study, and has been so laudably made mention of through your columns, fails to have one of the names of its faculty on the list of membership of our State Association.

The Chicago Police Department has 3 veterinary surgeons on its pay-roll, all graduates of recognized colleges, the names of neither appearing on the list of membership. The veterinary surgeons located at the Union Stock-yards, who are in connection with the Bureau of Animal Industry, take no interest in the Association.

You advise through the columns of your March number that the veterinary surgeons throughout the country should use their influence for the passage of the bills pending before Congress at Washington to give rank to veterinarians in the army. It behooves every veterinary surgeon in this country who has the dignity and honor of his profession at heart to become a member of the local association, so as to give more influence in their unity to any bill that is before either State or national legislative bodies. Very respectfully yours,

R. G. Walker, M.D.C.

Chicago, Ill.

---

### REQUEST FOR IMPORTANT INFORMATION.

Editor Journal of Comparative Medicine, etc.

For purpose of an extended study I request from veterinarians and others data as to habit, instinct, or intelligence of animals in health and disease, especially minor, trifling, apparently useless, nonsensical, or harmful habits. *Circular of information* will be sent, and full credit given or sender's name confidential, as preferred. Among habits desired are: Licking themselves, other animals or men; pairing and breeding habits; parturition; those connected with urine and feces (covering up, eating, etc.); disposition of feces or shells in nest; rolling on carrion; cackling (or other disturbance) after laying (does copulation follow?); eating afterbirth or young; sharing nest with or rearing young of another species with resulting modifications of instinct; hereditary transmission of peculiar traits; feigning death; "fascination;" transporting eggs or young, etc.

Answer *as fully* as possible, always stating age, sex, place, season, species, breed (pure?), and whether personally observed.

R. R. Gurley, M.D.

Clark University, Worcester, Mass.

